# Eosinophilic panniculitis presenting with Kaposi’s sarcoma-like plaques in a patient who is human immunodeficiency virus positive: a case report

**DOI:** 10.1186/1752-1947-6-387

**Published:** 2012-11-13

**Authors:** Pelin Ustuner, Nursel Dilek, Yunus Saral, Aziz Ramazan Dilek, Recep Bedir

**Affiliations:** 1 Rize State Hospital, Dermatology Clinic, Eminettin Caddesi, Hastaneler Kavşağı, Rize, 53100, Turkey; 2 Department of Dermatology, Recep Tayyip Erdoğan University School of Medicine, İslampaşa mahallesi, Tıp Fakültesi Dekanlığı, Rize, 53100, Turkey; 3 Department of Microbiology, Recep Tayyip Erdoğan University School of Medicine, İslampaşa mahallesi, Tıp Fakültesi Dekanlığı, Rize, 53100, Turkey; 4 Department of Pathology, Recep Tayyip Erdoğan University School of Medicine, İslampaşa mahallesi, Tıp Fakültesi Dekanlığı, Rize, 53100, Turkey

**Keywords:** Eosinophilic panniculitis, Human immunodeficiency virus, Kaposi’s sarcoma, Panniculitis

## Abstract

**Introduction:**

Eosinophilic panniculitis is an unusual type of panniculitis characterized by a prominent infiltration of subcutaneous fat with eosinophils without an exact etiopathogenesis. To the best of our knowledge, up to now eosinophilic panniculitis has been described in only one previous case with human immunodeficiency virus disease in the literature.

**Case presentation:**

Here we report the case of a 44-year-old Caucasian man, who is human immunodeficiency virus positive, diagnosed with eosinophilic panniculitis. A dermatological examination revealed multiple, confluent Kaposi’s sarcoma-like purple colored, deep plaques and nodules on his right gluteal area and right thigh. The presence of the mixed inflammatory infiltrate of lymphocytes, macrophages, and numerous eosinophils involving both septa and lobules of the subcutis were noted on the histopathological examination. On the basis of all these clinical and histopathological findings the patient was diagnosed with eosinophilic panniculitis. He was given intravenous 60mg/day methylprednisolone for 3 consecutive days a week for 6 months. The lesions resolved almost completely after 6 months.

**Conclusion:**

The predominance of T helper-2 subset of T helper cells and the consequential increase in interleukin-5 cytokines accompanying peripheral eosinophilia and high serum immunoglobulin E levels may all be blamed for the development of eosinophilic panniculitis in our case study. As a result, we aim to emphasize that eosinophilic panniculitis should be kept in mind in the differential diagnosis of subcutaneous nodular lesions in patients who are human immunodeficiency virus positive. We also focus on the requirement of histopathological examination for the definitive diagnosis because the clinical features of eosinophilic panniculitis may easily be confused with Kaposi’s sarcoma.

## Introduction

Eosinophilic panniculitis (EP) is an unusual type of panniculitis characterized by a prominent infiltration of subcutaneous fat with eosinophils [1]. It is a reactive pattern that may be associated with a variety of conditions such as arthropod bites, parasitic infestations, drug reactions, vasculitis, neoplasm, or hematologic diseases. The exact pathogenesis of the disease is still unclear. It is believed that in a patient with immunodeficiency due to hematologic disease a trigger-like insect bite, drug, or virus induces cytokine production with an excess of interleukin (IL)-4 and IL-5 and an altered immune response with eosinophil predominance [1]. In addition, in a patient with immunodeficiency the infiltration may be the result of an antigenic trigger like an insect bite, a drug, or a virus which causes cytokine production with an excess of IL-4 and IL-5 and an altered immune response with prominent infiltration of the septa and/or lobules of the subcutaneous fat with eosinophils leading to EP [1]. Here we report the case of a 44-year-old man, who has the human immunodeficiency virus (HIV), diagnosed with Kaposi’s sarcoma-like EP who responded well to corticosteroids.

## Case presentation

**Figure 1:**
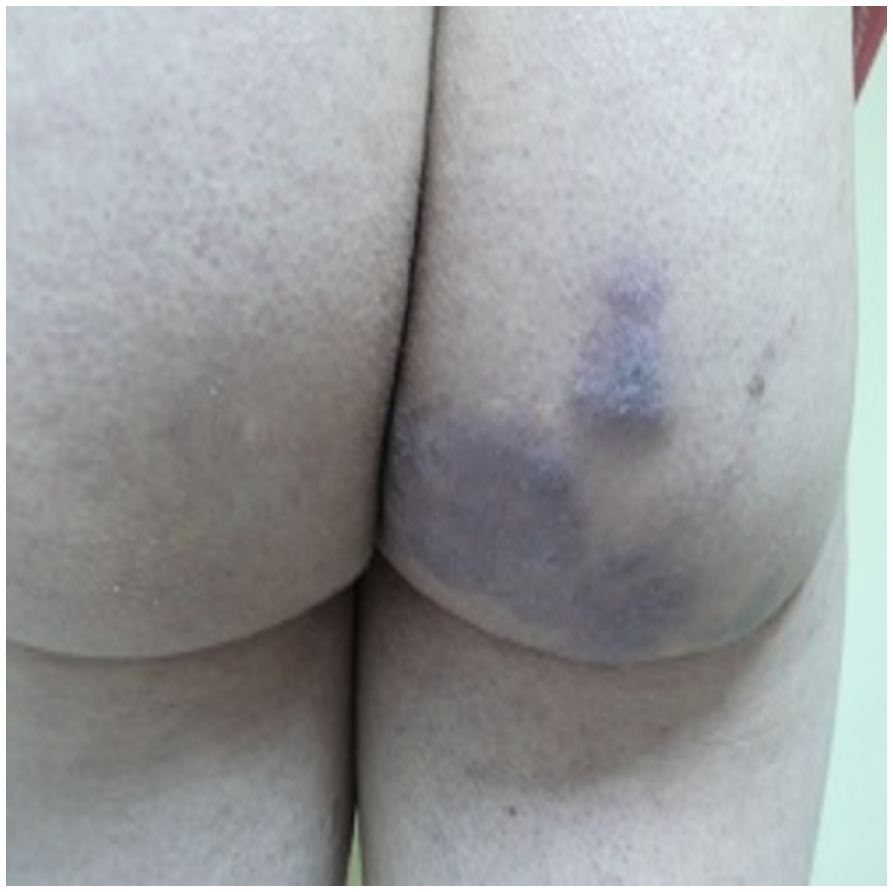
Multiple, confluent, Kaposi’s sarcoma-like purple colored, deep plaques and nodules on patient’s right gluteal area.

**Figure 2:**
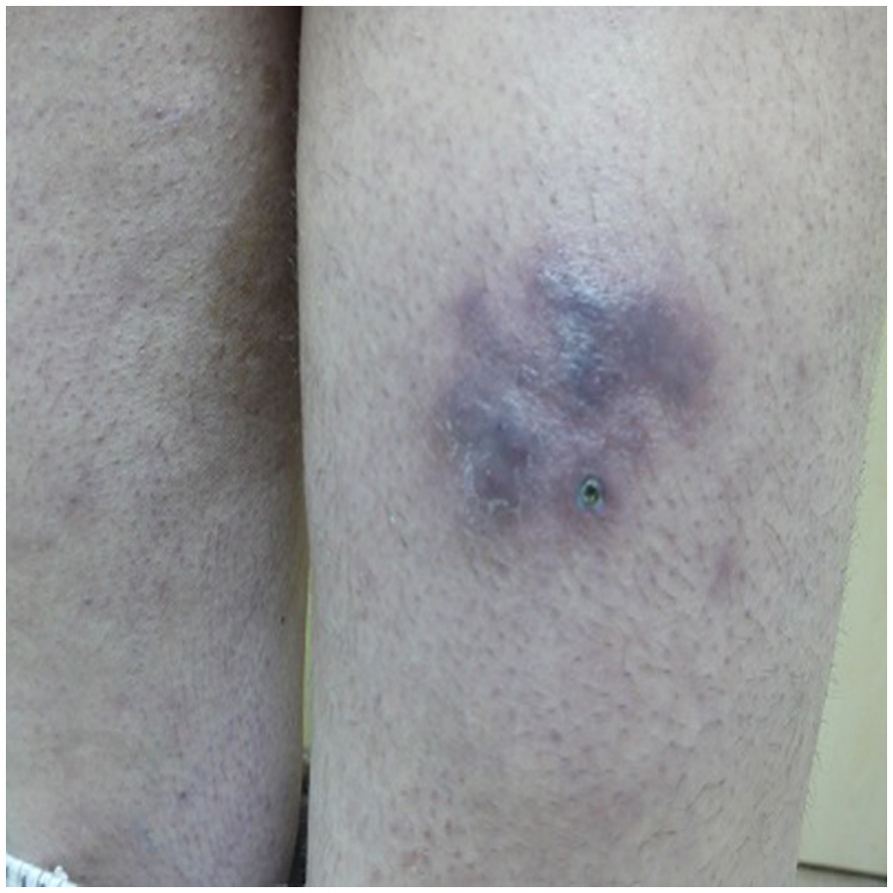
The irregular shaped plaques with a pebbled surface on patient’s right thigh.

**Figure 3:**
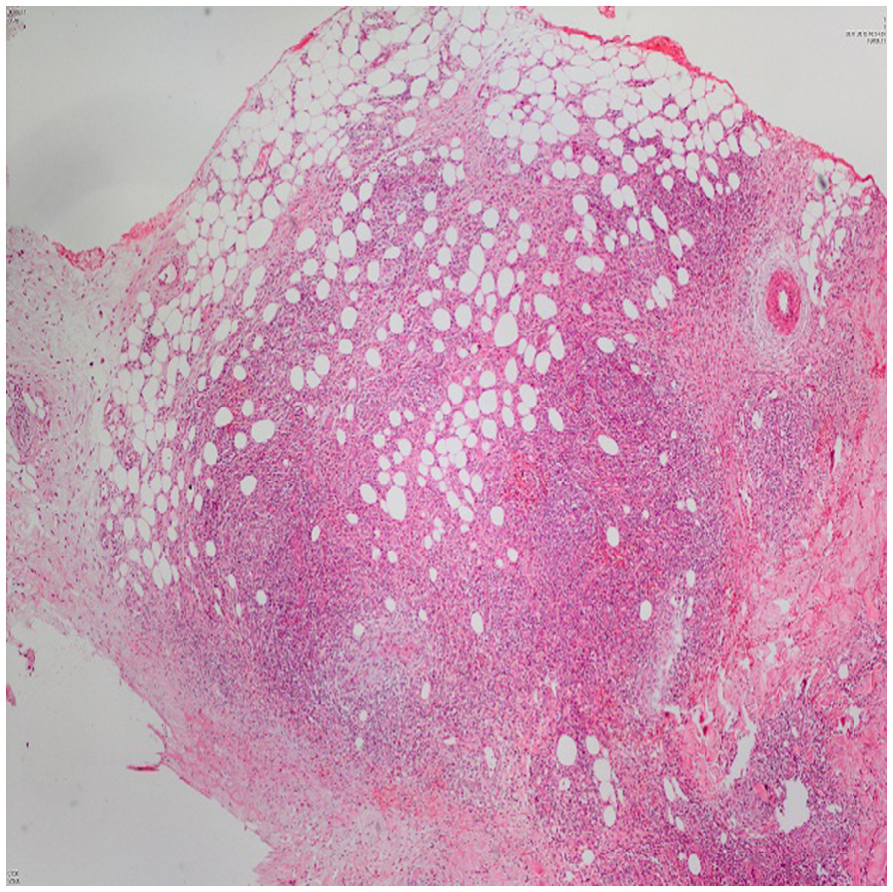
On the histopathological examination most of the inflammation was observed in the subcutis, in and near the septa and lobules (hematoxylin and eosin ×40).

**Figure 4:**
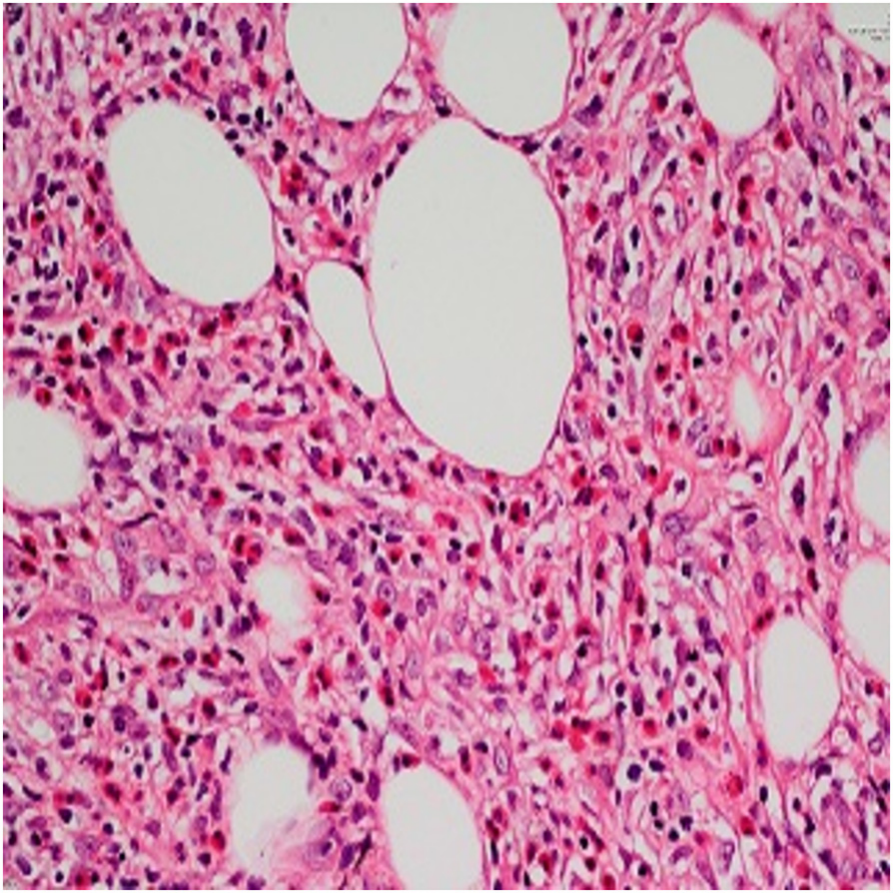
A mixed inflammatory infiltrate of lymphocytes, macrophages, and numerous eosinophils involving both septa and lobules of the subcutis (hematoxylin and eosin ×400).

A 44-year-old White Caucasian man who is HIV positive presented with multiple, pruritic, purplish colored plaques on the gluteal areas and thigh for 3 months. He was HIV positive for about 15 years. He was already at the stage of late symptomatic acquired immunodeficiency syndrome (AIDS) with a CD4 count of 350 cells/mm^3^ for 6 months. The HIV ribonucleic acid (RNA) titer was 7000 copies/mm^3^. His antiretroviral therapy included 600mg twice a day indinavir, 100mg twice a day ritonavir and 400mg lopinavir, 1200mg twice a day nelfinavir, 600mg per day zidovudine, and 150mg per day lamivudine. He had been treated with anti-HIV drugs for the past 6 months. He was also taking prophylactic azithromycin, dapsone, valacyclovir, gancyclovir and antifungals. His past medical and family history were unremarkable other than his HIV positivity. He denied the presence of a previous trauma, atopy or an arthropod bite. He also informed us that he had been treated with intravenous (IV) methylprednisolone in 0.3 to 0.5mg/kg doses a few weeks before without an exact histopathological diagnosis. A dermatological examination revealed multiple, confluent Kaposi’s sarcoma-like purple colored, deep plaques and nodules on his right gluteal area (Figure 1). There were also similar irregular shaped plaques with a pebbled surface on his right thigh (Figure 2). All the lesions were deeply extended through the subcutaneous tissue. On histopathological examination it was observed that most of the inflammation was in the subcutis, in and near the septa and lobules (hematoxylin and eosin stain ×40) (Figure 3). A mixed inflammatory infiltrate of lymphocytes, macrophages, and numerous eosinophils involving both septa and lobules of the subcutis were noted. There was a marked abundance of eosinophils in the infiltrate (Figure 4). The full blood examinations and urine analysis were all normal other than a mild lymphopenia (0.8 × 109/L). The sedimentation rate was increased (26mm/hour). Neither peripheral eosinophilia, nor an increase in total immunoglobulin E (IgE) level was observed. The serological tests including polymerase chain reaction were negative for *Toxoplasma gondii*, cytomegalovirus and human herpes virus-8. Hepatitis markers and Venereal Disease Research Laboratory tests were also negative. Direct radiographies and microbiological cultures excluded the presence of an infection, parasitic infestations, neoplasm and sarcoidosis. A punch biopsy sample was also sent for Gram stain, acid-fast bacillus staining, and fungal cultures, all of which were negative. With all these clinical and histopathological findings the patient was diagnosed with EP. He was given intravenous (IV) 60mg per day methylprednisolone for 3 consecutive days a week for 6 months. The lesions resolved almost completely after 6 months. No recurrence was noted during 6 months of follow-up.

## Discussion

EP is a histopathologic pattern rather than a distinct entity [1]. Previous clinical studies have already revealed diverse patterns of local and systemic diseases, including arthropod bites, Wells’ syndrome presenting with eosinophilic cellulitis, immune complex vasculitis, atopy, erythema nodosum, hematologic disorders like refractory anemia with excess blasts, injection granuloma, streptococcal and other bacterial infections, toxocariasis, malignancies like B- and T-cell lymphoma, polyarteritis nodosa, eosinophilic fasciitis, eosinophilic folliculitis, hypereosinophilic syndrome, eosinophilic neuritis, chronic recurrent parotitis, nephrotic syndrome, glomerulonephritis, sarcoidosis, tuberculous pleurisy, larva migrans (gnathostomiasis), and psychiatric illnesses like drug dependency [2-4]. The skin lesions mostly vary from urticarial papules, plaques to purpura, pustules, and ulcerative lesions but always include a nodular subcutaneous component [1]. Treatment is difficult and recurrences are common. It is a self-limited disorder in most cases, and has been shown to respond to prednisone, but lesions often recur when the dose is reduced. Chemotherapy regimens may also improve the lesions in some patients [1].

To the best of our knowledge, this is the second reported HIV positive case with EP described in the literature. The first case was a 32-year-old African American man with advanced AIDS who presented with generalized painful, pruritic papular rash, peripheral blood eosinophilia and perineural eosinophilic infiltrates with EP [5]. We propose that EP may represent additional findings in the spectrum of cutaneous diseases seen in patients with advanced AIDS. In later stages of HIV infection some immunologic abnormalities occur, such as a switch from predominately T helper-1 to T helper-2 subset of T helper cells that produce IL-5: a cytokine involved in eosinophil development and differentiation. The increase in IL-5 and IL-4 may be responsible for the possible eosinophilia and elevation of serum immunoglobulin E levels, respectively, in HIV-related eosinophilic diseases like eosinophilic folliculitis and EP [6]. However, we believe that the absence of peripheral eosinophilia in our case might also have been as a result of the previous corticosteroid treatments. In a recently reported comparative study, HIV-related pruritic papular eruption (HIV-PPE) and eosinophilic folliculitis were quantitatively distinguished mainly by the histopathological and immunohistochemical findings [7]. The intense perivascular and diffuse inflammatory infiltration, the tissue mast cell count by toluidine staining and expression levels of CD15 (for eosinophils), CD4 (T helper), and CD7 (pan-T lymphocytes) seen in HIV-EP were reported to be significantly higher than in HIV-PPE. The blood eosinophils were found to be low in both groups as in our case study. The difference of the percentage of the eosinophilic infiltrates was also reported to be nonsignificant [7]. The clinical similarity of the lesions to Kaposi’s sarcoma was also noteworthy in this case. Kaposi’s sarcoma was also firstly considered in the differential diagnosis because the patient was HIV positive and the lesions were vivid and purple colored. However, it was clearly excluded by the histopathological examinations.

## Conclusion

Although the etiology of EP is highly diverse and nonspecific, it seems to be a unique histopathological reaction pattern characterized by massive eosinophilic infiltration into subcutaneous tissues. Once a diagnosis of EP has been established, appropriate detailed evaluations for all the possible associated clinical conditions should be performed. EP should also be kept in mind in the differential diagnosis of the subcutaneous nodular lesions in patients who are HIV positive, and histopathological examination is mandatory for the definitive diagnosis. As a result, the predominance of T helper-2 subset of T helper cells and the consequential increase in IL-5 cytokines accompanying peripheral eosinophilia and high serum IgE levels may be blamed for the development of EP in patients who are HIV positive.

## Consent

Written informed consent was obtained from the patient for publication of this manuscript and any accompanying images. A copy of the written consent is available for review by the Editor-in-Chief of this journal.

## Abbreviations

AIDS: Acquired immunodeficiency syndrome; EP: Eosinophilic panniculitis; HIV: Human immunodeficiency virus; IL: Interleukin; PPE: Pruritic papular eruption.

## Competing interests

The authors declare that they have no competing interests.

## Authors’ contributions

All the authors have made substantive intellectual contributions to this study. We also want to emphasize that each author has participated sufficiently in the work to take public responsibility for appropriate portions of the content. PU, ND and YS have made substantial contributions to conception and design, or acquisition of data, or analysis and interpretation of data. The punch biopsy material was taken by ND. PU has been involved in drafting the manuscript or revising it critically for important intellectual content. RAD performed the serological and microbiological tests. RB analyzed the histopathological data. All authors read and approved the final manuscript.
